# Change in the direct cost of treatment for children and adolescents with hyperkinetic disorder in Germany over a period of four years

**DOI:** 10.1186/1753-2000-3-3

**Published:** 2009-01-28

**Authors:** Peter M Wehmeier, Alexander Schacht, Aribert Rothenberger

**Affiliations:** 1 Medical Department, Lilly Deutschland GmbH, Bad Homburg, Germany; 2 Department of Child and Adolescent Psychiatry, University of Göttingen, Göttingen, Germany

## Abstract

**Background:**

In many developed countries, the treatment of hyperkinetic disorder (or ADHD) consumes a considerable amount of resources. The primary aim of this study was to determine change in the direct cost of treatment for children and adolescents with hyperkinetic disorder in Germany over time, and compare the cost with the cost of treatment for two physical disorders: epilepsy and asthma.

**Methods:**

The German Federal Statistical Office provided data on the direct cost of treating hyperkinetic disorder, epilepsy and asthma in Germany for 2002, 2004, and 2006. The direct costs of treatment incurred by hyperkinetic disorder in these years were compared with those incurred by epilepsy and asthma.

**Results:**

The total direct cost of treatment for the hyperkinetic disorder was € 177 million in 2002, € 234 million in 2004, and € 341 million in 2006. The largest proportion of the cost was incurred by the age group < 15 years: € 158 million in 2002, € 205 million in 2004, and € 287 million in 2006. The direct cost of treatment for epilepsy in this age group was a total of € 157 million in 2002, € 155 million in 2004, and € 155 million in 2006. For asthma, the total direct cost of treatment in this age group was € 266 million in 2002, € 257 million in 2004, and € 272 million in 2006.

**Conclusion:**

The direct cost of treatment for hyperkinetic disorder in the age group < 15 years increased considerably between 2002 and 2006. Over the same period of time and for the same age group, expenditure for epilepsy and asthma was more or less constant. The increase in expenditure for the treatment of hyperkinetic disorder may be due to increasing demand for diagnostic and therapeutic services and improved availability of such services. The study is limited by the difficulty of obtaining consistent data on the direct cost of treatment for both physical and psychiatric disorders in Germany.

## Background

Hyperkinetic disorder (ICD-10) [1] or attention-deficit/hyperactivity disorder (DSM-IV-TR) [2] is one of the most common psychiatric disorders in childhood and adolescence. The disorder is characterized by the core symptoms attention deficit, hyperactivity and impulsivity. These core symptoms occur as a continuous pattern and are inappropriate relative to the child's age, developmental stage and intelligence. They occur consistently and in various situations (e. g. at school, at home in the family, or whilst together with peers) and lead to significant impairment in the child's cognitive and psychosocial level of functioning, emotional well-being and quality of life [3-6].

In school-age children, the prevalence of this disorder is reported to be 3–7% [2]. However, the prevalence rates found in various studies differ considerably depending on the particular classification system and diagnostic methods used [7]. The world-wide pooled prevalence has recently been reported to be 5.29% [8]. Boys are two to nine times more commonly affected than girls [2]. Hyperkinetic disorder may interfere with the daily life of patients and their families to a greater degree than physical disorders such as asthma [9,10]. The long-term consequences of ADHD include loss of productivity, healthcare consumption, material damage, criminality, lost life years, intangible and other costs [10,11].

Hyperkinetic disorder is usually treated using a multi-modal treatment plan that may include interventions such as behavioural therapy, parent counseling and/or medication [12-16]. In severe cases, hospitalization may be necessary. In Germany, hospitalization rates vary from region to region, depending on the availability of outpatient treatment opportunities. In regions with a low density of office-based physicians, hospitalization rates are higher than in regions with a high density of office-based physicians [17]. Treatment of hyperkinetic disorder results in substantial use of health care resources [10], on one hand through the cost incurred on payers such as health insurance providers, on the other through the additional financial burden placed on patients and their families [18].

In many developed countries, the treatment of hyperkinetic disorder (or ADHD) consumes a considerable amount of resources. Attempts at determining the cost of ADHD and the cost-effectiveness of various treatments for ADHD have led to a range of results, some of which concur whilst others are contradictory [14,15,17,19-25]. However, there is general agreement that ADHD has a considerable impact both on direct and indirect costs caused by the disorder. In health economic assessments, costs are usually divided into direct and indirect costs [11]. In these assessments, direct costs refer to consumption of resources as a direct consequence of the disorder (e. g. medical treatment), whilst indirect costs refer to indirect consequences due to the disorder (e. g. the inability to do work) and the resulting costs to society due to loss of productivity. Whilst the direct costs of a disorder are relatively easy to determine, the assessment of the indirect costs may require the use of more or less elaborate socio-economic models and calculations [11,17,18,26-37].

Empirical data on the direct cost of treatment for hyperkinetic disorder have not been available for Germany. Therefore, the main objective of this analysis was to determine the direct cost of treatment (CoT) associated with hyperkinetic disorder in children and adolescents in Germany, broken down by age and sex, and compare the findings with the direct CoT of two fairly common physical disorders in childhood, namely epilepsy and asthma, since such a comparison is demanded by public health politicians in order to discuss allocation of financial resources. A further objective of this analysis was to identify any changes in the direct CoT over time. Based on the considerable increase in methylphenidate prescriptions as well as increasing availability of evidence based behavioural treatment programs and more inpatient and outpatient treatment opportunities in Germany in the 1990s [38], our expectation was that the total direct cost of treatment would be seen to increase further over time, whilst the increase in the total direct cost of treatment for epilepsy and asthma would be much lower.

## Methods

Data on the total direct cost of treatment for hyperkinetic disorder, epilepsy and asthma were provided by the German Federal Statistical Office (Statistisches Bundesamt) for the years 2002, 2004, and 2006. These data are collected on an annual basis by the Statistical Office from health insurance providers and reported in summarized form on a bi-annual basis [39]. The data reflect the cost of treatment very well, as the data are based on the actual expenditure of the health insurance providers [40]. The Federal Statistical Office uses a top-down approach based on data from hospitals, physicians' offices, rehabilitation units, pharmacies etc. ("Krankheitskostenrechnung"). In the bi-annual report, the data are broken down by diagnosis (in this case hyperkinetic disorder, epilepsy, and asthma), age (age groups < 15 years, 15–30 years, 30–45 years, 45–65 years, 65–85 years, and over 85 years), sex, and the various types of treatment (inpatient treatment, outpatient treatment, medication, other treatments). "Inpatient treatment" comprises hospital care and treatment provided in a rehabilitation unit, "outpatient treatment" comprises treatment by office-based physicians and the cost of outpatient nursing care, "medication" comprises cost for medication provided by retail pharmacies (excluding hospital pharmacies), and "other treatment costs" comprise any other direct cost ultimately reimbursed by health insurance providers such as medical emergency services, auxiliary medical services, treatment provided in a foreign country, or administrative costs. The methodology on which the report is based accounts for the primary diagnosis only and not for comorbid disorders. However, if two equally important diagnoses are reported, the costs of treatment are split equally among the two diagnoses.

As the data in the bi-annual report are not detailed enough to carry out a comparison between hyperkinetic disorder, epilepsy, and asthma in terms of direct cost of treatment, we requested a sub-analysis from the German Federal Statistical Office that allows this comparison. Based on this sub-analysis, we compared descriptively the direct cost of treatment for hyperkinetic disorder with the direct cost of treatment for epilepsy and asthma.

## Results

The total direct cost of treatment for the hyperkinetic disorder was € 177 million in 2002, € 234 million in 2004, and € 341 million in 2006. The largest proportion of the cost was incurred by the age group < 15 years: € 158 million in 2002, € 205 million in 2004, and € 287 million in 2006. The direct cost of treatment for epilepsy in this age group was a total of € 157 million in 2002, € 155 million in 2004, and € 155 million in 2006. For asthma, the total direct cost of treatment in this age group was € 266 million in 2002, € 257 million in 2004, and € 272 million in 2006 (Table 1). As expected, the total direct cost of treatment increased over time, whilst the change in the total direct cost of treatment for epilepsy and asthma over the same time period was negligible.

**Table 1:** Direct cost of treatment for hyperkinetic disorder, epilepsy, and asthma in Germany for the age group < 15 years, shown by diagnosis and type of treatment.

**Diagnosis/**Treatment	**Total cost of treatment for 2002 in millions**	**Total cost of treatment for 2004 in millions**	**Total cost of treatment for 2006 in millions**

**Hyperkinetic disorder**	€ 158 (100%)	€ 205 (100%)	€ 287 (100%)

Inpatient treatment	€ 73 (46.2%)	€ 93 (45.4%)	€ 112 (39.0%)

Outpatient treatment	€ 12 (7.6%)	€ 14 (6.8%)	€ 21 (7.3%)

Medication (outpatients)	€ 12 (7.6%)	€ 28 (13.7%)	€ 81 (28.2%)

Other treatment costs	€ 61 (38.6%)	€ 70 (34.2%)	€ 73 (25.4%)

**Epilepsy**	€ 157 (100%)	€ 155 (100%)	€ 155 (100%)

Inpatient treatment	€ 74 (47.1%)	€ 73 (47.1%)	€ 73 (47.1%)

Outpatient treatment	€ 9 (5.7%)	€ 8 (5.2%)	€ 9 (5.8%)

Medication (outpatients)	€ 22 (14.0%)	€ 24 (15.5%)	€ 24 (15.5%)

Other treatment costs	€ 52 (33.1%)	€ 50 (32.3%)	€ 49 (31.6%)


**Asthma**	€ 266 (100%)	€ 257 (100%)	€ 272 (100%)

Inpatient treatment	€ 92 (34.6%)	€ 83 (32.3%)	€ 82 (30.1%)

Outpatient treatment	€ 27 (10.2%)	€ 32 (12.5%)	€ 40 (14.7%)

Medication (outpatients)	€ 103 (38.7%)	€ 98 (38.1%)	€ 105 (38.6%)

Other treatment costs	€ 44 (16.5%)	€ 44 (17.1%)	€ 45 (16.5%)

Approximately two thirds of the patients in this sample treated for hyperkinetic disorder are in the age group < 15 years [41]. In 2002, a total of € 128 million was incurred by boys, and € 31 million by girls in this age group. Proportions were similar in the following years: in 2004 a total of € 167 million were incurred by boys and € 38 million by girls, and in 2006 a total of € 237 million was incurred by boys and € 50 million by girls.

The greatest proportion of these costs resulted from inpatient treatment. In 2002, € 73 million (46.2% of the total direct cost of treatment) resulted from inpatient treatment, whilst € 93 million (45.4% of the total direct cost of treatment) resulted from inpatient treatment in 2004, and € 112 million (39.0% of the total direct cost of treatment) resulted from inpatient treatment in 2006. A smaller proportion of the total cost resulted from outpatient treatment, medication, and other treatment costs (Table 1).

The total direct cost of treatment resulting from patients with epilepsy in the age group < 15 years was € 157 million in 2002, € 155 million in 2004, and € 155 million in 2006. The greatest proportion of these costs resulted from inpatient treatment. The total direct cost of treatment for asthma in the age group < 15 years was € 266 million in 2002, € 257 million in 2004, and € 272 million in 2006. The largest proportion of these costs were cost of medication (Table 1).

The direct cost of treatment for hyperkinetic disorder, epilepsy, and asthma for the age group < 15 years and for the years 2002, 2004 and 2006 is shown separately for males and females in Table 2. The cost-ratio males to females corresponds to the epidemiological-ratio of approximately 4:1.

**Table 2:** Direct cost of treatment for hyperkinetic disorder, epilepsy, and asthma in Germany for the age group < 15 years, shown by type of cost and sex.

**Treatment/**Diagnosis	**Cost of treatment for 2002 in millions**		**Cost of treatment for 2004 in millions**		**Cost of treatment for 2006 in millions**	
	males	females	males	females	males	females

**Inpatient treatment**						

Hyperkinetic disorder	€ 60	€ 12	€ 78	€ 15	€ 94	€ 18

Epilepsy	€ 39	€ 35	€ 39	€ 34	€ 39	€ 33

Asthma	€ 58	€ 34	€ 53	€ 30	€ 51	€31


**Outpatient treatment**						

Hyperkinetic disorder	€ 9	€ 2	€ 10	€ 3	€ 16	€ 5

Epilepsy	€ 5	€ 4	€ 4	€ 4	€ 4	€ 4

Asthma	€ 17	€ 10	€ 20	€ 12	€ 26	€ 14


**Medication (outpatients)**						

Hyperkinetic disorder	€ 10	€ 2	€ 23	€ 5	€ 67	€ 13

Epilepsy	€ 13	€ 9	€ 12	€ 11	€ 14	€ 11

Asthma	€ 67	€ 36	€ 62	€ 36	€ 68	€ 37


**Other treatment costs**						

Hyperkinetic disorder	€ 49	€ 15	€ 56	€ 15	€ 60	€ 14

Epilepsy	€ 29	€ 23	€ 30	€ 21	€ 28	€ 22

Asthma	€ 28	€ 16	€ 27	€ 17	€ 27	€ 18

## Discussion

The total direct cost of treatment (CoT) for hyperkinetic disorder in the age group < 15 years in 2002 in Germany was € 177 million. In 2004, the total direct CoT was € 234 million, and in 2006 € 341 million. This considerable increase in the total direct CoT may be explained by more extensive use of opportunities to diagnose and treat the disorder, resulting in a greater number of children and adolescents being treated. Another possible explanation is that treatment is increasingly becoming evidence-based and guideline oriented, resulting in longer courses of treatment and greater amounts of medication being prescribed. It is remarkable, that the cost of medication more than doubled between 2002 and 2004, and more than doubled again between 2004 and 2006. This marked increase in the resources spent on medication to treat hyperkinetic disorder corresponds to earlier findings that showed a marked increase in prescriptions of methylphenidate in the 1990s in Germany [38]. However, the cost resulting from inpatient treatment also increased. Comparing these costs with the cost of treatment for epilepsy or asthma shows that the costs incurred by treating these two physical disorders remained fairly stable over the same period of time. This applies to all types of treatment (inpatient treatment, outpatient treatment, medication, other treatment costs). The increase in costs for inpatient treatment for hyperkinetic disorder may be explained by improved treatment opportunities, better treatment facilities with greater treatment capacities, new and effective treatment approaches, and increasing awareness of hyperkinetic disorder as a challenge to public health in Germany.

This marked increase in the cost of treatment for hyperkinetic disorder has resulted in hyperkinetic disorder overtaking asthma as the disorder with the greatest total direct cost in the age group < 15 years in the year 2006. This was not the case in 2002 and 2004, when asthma was the disorder with the greatest total direct cost in this age group by a considerable margin.

As might be expected in face of the different prevalence of hyperkinetic disorder in boys and girls, the total direct cost due to the treatment of boys is indeed higher than the cost due to the treatment of girls with hyperkinetic disorder (Table 2) and indicates that girls with ADHD need similar financial resources as boys.

The data provided by the German Federal Statistical Office on total direct cost of treatment can be compared with data from other sources. One such source is the annual report on prescriptions in Germany (Arzneiverordnungsreport, GKV-Arzneimittelindex, Wissenschaftliches Institut der AOK) that provides data on the number of prescriptions reimbursed by public health care providers, which comprise approximately 90% of all patients (the remaining 10% being privately insured). With this approach, the number of prescriptions is multiplied by the cost of one Defined Daily Dose (DDD) for a particular medication in order to arrive at the direct cost of medication for a particular disorder. These reports also show a marked increase in expenditure for medication used to treat hyperkinetic disorder, mainly methylphenidate: € 23.7 million in 2002, € 51.4 million in 2004, and € 108.8 million in 2006 [42-44]. Whilst this trend closely resembles the trend demonstrated by data from the German Federal Statistical Office, there are several discrepancies in terms of the direct cost resulting from medication for hyperkinetic disorder. However, the discrepancies can be explained by methodological differences between the approaches. The annual reports on prescriptions in Germany have several limitations. First, the data only reflect the cost incurred by 90% of the patients. Secondly, the annual reports do not break down the costs by age. This means that adults who receive methylphenidate prescriptions cannot be distinguished from children and adolescents who receive similar prescriptions. Thirdly, the data reflect costs incurred by a particular compound rather than a particular disorder. Thus, the annual reports on prescriptions reflect medication-related cost, whilst the data provided by the German Federal Statistical Office reflect disorder-related cost. As a given compound may have more than one indication (e. g. methylphenidate for both hyperkinetic disorder and narcolepsy), the annual reports do not allow clear distinction between disorders that happen to be treated with the same medication. In turn, a given disorder may require treatment with several different compounds, as is commonly the case in epilepsy or asthma.

The data provided by the German Federal Statistical Office on total direct cost of treatment also has several limitations due to the methodology by which they are collected and analyzed. Although the data reflect the cost incurred by 100% of the patients (both those with public and private health insurance), the costs are broken down by the following age groups: < 15 years, 15–30 years, 30–45 years, 45–65 years, 65–85 years, and over 85 years. As a result, adolescents ≥ 15 years of age are in one group with young adults. However, as the number of adolescents treated for hyperkinetic disorder decreases dramatically with age [41], the great majority of patients on medication are in the age group < 15 years, with only a very small number of adolescents ≥ 15 years of age being treated for this disorder. These methodological differences in the approaches explain the discrepancies in the two data sets.

This study has a number of limitations. Due to the type of data collected by the German Federal Statistical Office and the sources of these data (health insurance providers), the data only reflect the direct cost of treatment, not the total cost of hyperkinetic disorder to society. Another limitation relates to the observation period. Because data were available only for the years 2002, 2004, and 2006, it was not possible to track the change in cost of treatment over a longer period of time. Furthermore, it would have been interesting to compare the direct cost of treatment for hyperkinetic disorder with a broader range of both physical and psychiatric disorders. However, due to the limited data available from the Statistical Office, the cost for treating hyperkinetic disorder could only be compared with the cost for treating epilepsy and asthma. Finally, since the study was carried out on the basis of data from the German health care system, it is difficult to relate these findings to the direct cost of treatment in other countries. However, in the absence of other comparative data and considering the difficulties involved in obtaining such data, the findings from this study provide a rough approximation of the total direct cost of treatment for hyperkinetic disorder compared to epilepsy and asthma in an industrialized country in Western Europe.

The increase in the direct cost of treatment for hyperkinetic disorder runs parallel with recent improvement of diagnostic capabilities and treatment options [14,45]. In addition to child and adolescent psychiatrists, paediatricians and general practitioners increasingly treat children and adolescents with hyperkinetic disorder, one important reason being that child and adolescent psychiatrists alone are unable to meet the demand for all patients seeking diagnostic assessment and treatment. Therefore, the increase in cost incurred by medication for hyperkinetic disorder is not surprising, as it reflects years of unmet need [38,41] and indicates that an increasing number of children and adolescents with hyperkinetic disorder are now receiving an effective treatment. However, it remains to be seen whether this increase in cost of treatment will continue at the present rate, especially since more expensive long-acting medications have been introduced [14], or whether costs will cease to increase as the number of treated patients approaches the prevalence of hyperkinetic disorder. In any case, the challenge of optimizing and delivering cost-effective treatment for the individual patient remains [45]. Two consensus-conferences have resulted in the establishment of a central network for ADHD in Germany. This network involves child and adolescent psychiatrists, pediatricians, adult psychiatrists and clinical psychologists [46]. Although such interdisciplinary programs may potentially contribute to a further increase the direct cost of treating hyperkinetic disorder, improved treatment networks are likely to lead to a reduction in the indirect cost of the disorder, too. The direct and indirect costs of treatment are likely to develop inversely, thus reducing the total cost of the disorder to society in the long run. Full economic evaluation would have to be based on an analysis of cost-effectiveness (e. g. quality-adjusted life years) and would require further data.

## Conclusion

In summary, the results of this analysis support our expectation that the total direct cost of treatment would increase over time, whilst the increase in the total direct cost of treatment for epilepsy and asthma would be much lower. This shows that the gap caused by under-diagnosis and under-treatment of hyperkinetic disorder in Germany is closing. From a clinical point of view, this finding is encouraging.

## Competing interests

PMW and AS are full-time employees of Lilly Deutschland GmbH and are stock shareholders in Eli Lilly and Company. AR has received research support from Lilly Deutschland GmbH and is on several Lilly advisory boards.

## Authors' contributions

PMW conceived and designed the study, acquired the data, analyzed and interpreted the data, drafted the manuscript, and gave final approval of the version to be published. AS analyzed and interpreted the data, drafted the manuscript, and gave final approval of the version to be published. AR analyzed and interpreted the data, revised the manuscript, and gave final approval of the version to be published.
